# Emerging neurotrophic role of GABA_B_ receptors in neuronal circuit development

**DOI:** 10.3389/fncel.2013.00206

**Published:** 2013-11-12

**Authors:** Jean-Luc Gaiarsa, Christophe Porcher

**Affiliations:** ^1^Institut National de la Santé et de la Recherche Médicale U-901Marseille, France; ^2^Aix-Marseille Université, UMR S901Marseille, France; ^3^Institut de Neurobiologie de la MéditerranéeMarseille, France

**Keywords:** GABA_B_ receptors, GABA, cortical development, synaptogenesis, migration

## Abstract

The proper development of highly organized structures in the central nervous system is a complex process during which key events – neurogenesis, migration, growth, differentiation, and synaptogenesis – have to take place in an appropriate manner to create functional neuronal networks. It is now well established that GABA, the main inhibitory neurotransmitter in the adult mammalian brain, plays more than a classical inhibitory role and can function as an important developmental signal early in life. GABA binds to chloride-permeable ionotropic GABA_A_ receptors and to G-protein-coupled GABA_B_ receptors (GABA_B_-Rs). Although most of the trophic actions of GABA have been attributed to the activation of GABA_A_ receptors, recent advances show that GABA_B_-Rs also regulate fundamental steps of network development. This review summarizes some of the recent progress about the neurotrophic role of GABA_B_-Rs to neuronal development.

## INTRODUCTION

Gamma-aminobutyric acid (GABA) is the main inhibitory transmitter in the adult mammalian brain. GABAergic interneurons regulate neuronal excitability, synaptic integration, and the dynamic of network oscillations and as such are important for many cognitive functions. Recent advances, however, indicate that GABA can act beyond its classical role in synaptic communication and may function as an important developmental signal, being able to modulate nearly all key steps of neuronal network formation including cell survival and migration, neuritic growth and guidance, synapse formation and maturation (Owens and Kriegstein, 2002; Ben-Ari et al., 2007; Sernagor et al., 2010).

Most of the trophic actions of GABA have been attributed to the activation of the ionotropic, chloride permeable, GABA_A_ receptors. Indeed, early in development, GABA_A_ receptors activation induces a membrane depolarization and an increase in intracellular calcium concentration (Owens and Kriegstein, 2002; Ben-Ari et al., 2007). The depolarizing to hyperpolarizing shift of GABA_A_ receptor-mediated response results from a developmental decrease in intracellular chloride concentration brought about by the increased contribution of the potassium/chloride co-transporter, KCC2, which extrudes chloride from the cells (Rivera et al., 1999). The role of GABA_A_ receptors in neuronal development has been highlighted in studies in which the depolarizing/hyperpolarizing conversion of GABA responses is modified *in vitro* (Chudotvorova et al., 2005) and *in vivo* (Ge et al., 2006; Cancedda et al., 2007; Reynolds et al., 2008; Wang and Kriegstein, 2008). However, GABA also activates metabotropic GABA_B_ receptors (GABA_B_-Rs) and accumulating evidence indicate that these receptors may promote cell migration, differentiation, and synaptogenesis. The goal of this review is to recapitulate the current knowledge about the overlooked role of GABA_B_-Rs in neuronal development.

## A SHORT INTRODUCTION TO THE BASIC PROPERTIES OF GABA_**B**_-R SIGNALING

GABA_B_ receptors are metabotropic receptors coupled to G_α__i/o_-guanosine triphosphate (GTP) type protein which inhibits adenylate cyclase and gates ion channels (Bowery, 1993; Bowery et al., 2002). Released GABA can feed back onto GABA_B_ auto-receptors located on GABAergic terminals, and/or spillover to activate hetero-synaptic GABA_B_-Rs on neighboring glutamatergic terminals. Activation of the pre-synaptic GABA_B_-Rs inhibits neurotransmitter release through multiple targets including inactivation of voltage-dependent calcium channels (Mintz and Bean, 1993), gating of potassium conductance to shunt pre-synaptic action potentials (Thompson and Gahwiler, 1992a), reduction of vesicle priming (Sakaba and Neher, 2003), or interaction with the exocytosis machinery (Blackmer et al., 2005). Released GABA also signals onto post-synaptic GABA_B_-Rs located on dendritic shaft and spines (Kulik et al., 2003). Activation of these receptors generates slow (100-150 ms) inhibitory potentials via the opening of G-protein activated-inward rectifying potassium channels (G-protein-regulated inwardly rectifying K^+^ channels, GIRKs also known as inwardly rectifying potassium, Kir3 channels; Gähwiler and Brown, 1985).

The cloning of GABA_B_-Rs in the late 1990s has led to the identification of two GABA_B_ gene products: the GABA_B1_ and GABA_B2_ subunits (Kaupmann et al., 1997). Recombinant experiments showed that heterodimerization of GABA_B1_ and GABA_B2_ subunits is mandatory for cell surface expression and coupling to G-protein (Jones et al., 1998; Kaupmann et al., 1998; White et al., 1998). Coiled–coil interactions in the C-terminal domain of the newly synthesized subunits in the endoplasmic reticulum masks a retention signal present on the C-terminal domain of the GABA_B1_ subunit so that only GABA_B1_ subunit assembled with GABA_B2_ subunit are trafficked to the cell surface. GABA_B1_/GABA_B2_ subunits assembly is also mandatory for agonist-induced signaling. In the heterodimeric GABA_B_-Rs, GABA_B1_ subunit is responsible for binding of GABA, whereas the GABA_B2_ subunit is necessary for G-protein coupling (Robbins et al., 2001). Transgenic mice lacking the GABA_B1_ subunit confirm that heterodimeric assembly is required to provide fully functional receptors *in vivo* since GABA_B1_^-^^/^^-^ mice do not exhibit detectable electrophysiological, biochemical, or behavioral responses to GABA_B_-R agonists (Prosser et al., 2001; Schuler et al., 2001; Queva et al., 2003). Deletion of the GABA_B2_ subunit also abolished all known response to GABA_B_-R agonists (Gassmann et al., 2004). The GABA_B2_^-^^/^^-^ mice, however, exhibit an atypical baclofen response, namely an inhibition of potassium channels, which is not observed in wild type (WT) mice (Gassmann et al., 2004). Thus GABA_B1_ subunits could assemble into functional receptor but such homomeric assembly may be a consequence of the knockout of the GABA_B2_ subunit (Gassmann et al., 2004).

The GABA_B1_ subunit further exists under two isoforms, named GABA_B1a_ and GABA_B1b_, which differ by a pair of sushi domains on the N-terminal of the GABA_B1a_ subunit (Kaupmann et al., 1997; Biermann et al., 2010). The two isoforms have similar pharmacological and physiological properties in heterologous expression systems precluding determination of the functional significance of this molecular diversity. The demonstration that the GABA_B1a_ and GABA_B1b_ isoforms contribute to distinct native GABA_B_-Rs and convey different functions was made possible by the generation of mice deficient in GABA_B1a_ or GABA_B1b_ isoform. Using this knocking down approach, it was shown that the GABA_B1a_ isoform is preferentially targeted to the pre-synaptic glutamatergic terminals and assemble with GABA_B2_ subunit to form hetero-receptors whereas both GABA_B1a_ and GABA_B1b_ isoforms assemble with the GABA_B2_ subunit into auto-receptors at pre-synaptic GABAergic terminals (Vigot et al., 2006; Guetg et al., 2009). On the post-synaptic side, although both isoforms are present, GABA_B1b_ isoform provides the majority of coupling with GIRK (Vigot et al., 2006) and inhibition of dendritic calcium spikes (Perez-Garci et al., 2006).

## ONTOGENY OF GABA_B_ RECEPTOR-MEDIATED RESPONSES

Information regarding the distribution pattern and subcellular distribution of the GABA_B_ receptor subunits is crucial to gain insight into the contribution of these receptors to brain development. Several evidences indicate that both GABA_B1_ and GABA_B2_ subunits are expressed in the developing brain although some temporal and spatial differences exist, suggesting that the regulatory mechanisms for these subunits may differ. At the embryonic day (E) 11, GABA_B1_ transcripts are detected in the rat brain whereas the GABA_B2_ transcripts are below the level for detection (Kim et al., 2003; Lopez-Bendito et al., 2004; Martin et al., 2004). At E14, both transcripts are detected in most brain regions except in the olfactory bulb and striatum where GABA_B2_ transcripts remain barely detectable until E17 (Martin et al., 2004). At the protein levels, in a pioneer study, Turgeon and Albin (1994) performed quantitative receptor autoradiography with (^3^H)GABA to study the ontogeny of GABA_B_ binding sites in the rat brain. They reported that GABA_B_ binding levels is detected at early postnatal stages and peak at regionally specific times during the three first postnatal week of life. Western blot and immuno-histochemical analysis confirm that GABA_B1_ and GABA_B2_ subunits are present in the developing brain (Behar et al., 2001; Lopez-Bendito et al., 2002, 2004; Martin et al., 2004; Bianchi et al., 2005; Lujan and Shigemoto, 2006). These studies further show distinct expression patterns between GABA_B1a_ and GABA_B1b_ subunits (Fritschy et al., 1999; Fritschy et al., 2004). The GABA_B1a_ subunit is highest during the first postnatal week of life, whereas GABA_B1b_ subunit progressively increases to reach its maximum by P10 (Fritschy et al., 1999). Both subunits reach adult levels by the end of the third postnatal week of life. The expression of the GABA_B1_ and GABA_B2_ subunits overlaps in many regions but some laminar and cellular distinctions are found. For instance at birth, the GABA_B1_ labeling is uniform across all neocortical layers, while the GABA_B2_ labeling is most intense in the layers I and V–VI (Fritschy et al., 2004). Similarly, in the hippocampus, GABA_B1_ expression predominates in the pyramidal layer while GABA_B2_ is mostly expressed in the dendritic layers (Fritschy et al., 2004). In the gerbil medial superior olive (MSO), the GABA_B1_ expression changes from a predominantly dendritic to a somatic location during development (Hassfurth et al., 2010). Double immuno-labeling indicate that the GABA_B1_ and GABA_B2_ subunits co-localize in neurons of the marginal zone and subplate, while tangentially migrating neurons in the intermediate zone (iz) and Cajal–Retzus cells in the layer I only express the GABA_B1_ subunit (Lopez-Bendito et al., 2002). At the electron microscopic level, the GABA_B1_ and GABA_B2_ subunits are found at both pre- and post-synaptic levels in the developing cerebellum (Lujan and Shigemoto, 2006), neocortex (Lopez-Bendito et al., 2002), and hippocampus (Lopez-Bendito et al., 2004) at early postnatal development stages. At the post-synaptic levels, both subunits are present on dendritic shaft and spines at extra-synaptic and peri-synaptic sites. Of note is the transient expression of GABA_B1_ and GABA_B2_ subunits on glial cells in the hippocampus (Lopez-Bendito et al., 2004) and cerebellum (Lujan and Shigemoto, 2006). Activation of these receptors induce calcium transient in astrocytes in newborn rat hippocampal slices (Meier et al., 2008) that may in turn impact the development of neuronal networks.

Studies investigating the ontogeny of GABA_B_-R mediated responses further indicate that GABA_B_-Rs are present and functional early in development albeit some regional distinctions in the signaling pathway activated. Post-synaptic GABA_B_-mediated opening of GIRK to baclofen applications are not recorded at birth in the hippocampus (Gaïarsa et al., 1995; Caillard et al., 1998; Nurse and Lacaille, 1999; Verheugen et al., 1999) and neocortex (Luhmann and Prince, 1991; Fukuda et al., 1993; Kirmse and Kirischuk, 2006), while pre-synaptic GABA_B_-R mediated inhibition is already functional and controls both GABA and glutamate release (Luhmann and Prince, 1991; Fukuda et al., 1993; McLean et al., 1996; Caillard et al., 1998). Post-synaptic GABA_B_-mediated responses increase until the middle of the second postnatal week and remain stable thereafter (Luhmann and Prince, 1991; Fukuda et al., 1993; Gaïarsa et al., 1995; Kirmse and Kirischuk, 2006). Hippocampal neuronal cultures from E18 rat embryos also lack of post-synaptic GABA_B_-R mediated currents until 11 days *in vitro* (Ehrengruber et al., 1997) although they express both GABA_B1_ and GABA_B2_ subunits (Martin et al., 2004). When transfected to overexpress GIRK, a typical GABA_B_-R mediated potassium outward current could be induced in these neurons (Ehrengruber et al., 1997), suggesting that the availability of the GIRK channels is the likely limiting factor for the appearance of functional post-synaptic GABA_B_-R mediated inhibition (but see Correa et al., 2004).

Other evidence confirm that the post-synaptic GABA_B_-Rs are present and interact with G-proteins at early developmental stages. For instance, GABA_B_-R agonists decrease forskolin-induced cyclic-adenosine monophosphate (cAMP) levels in E18 neocortical neuronal cultures (Martin et al., 2004; Bony et al., 2013) and increase intracellular calcium concentration in some dissociated embryonic cortical neurons (Behar et al., 1996). In the developing rat hippocampus, baclofen induces a rapid increase of protein kinase C (PKC) activity (Tremblay et al., 1995). Patch-clamp recordings performed on developing chick retina have shown that GABA_B_-Rs failed to activate potassium currents, while they inhibit calcium channels (Catsicas and Mobbs, 2001). Similarly, in the developing rat hypothalamus baclofen depresses the post-synaptic calcium rise induced by glutamate- and GABA_A_-R agonists (Obrietan and Van den Pol, 1998, 1999). A completely different situation has been observed in the gerbil MSO where GIRK currents activated by post-synaptic GABA_B_-Rs disappear during development (Hassfurth et al., 2010). How this developmental change in GABA_B_-R mediated responses is regulated in presently unknown.

Altogether, these observations show that post-synaptic GABA_B_-Rs are present and functional early in development, although they are not yet involved in the control of cell excitability via the opening of post-synaptic GIRK in most developing brain regions. At that stage, they are, however, coupled to signaling pathways that may instruct or modulate neuronal development.

## THE CONDITIONS FOR THE ACTIVATION OF GABA_B_-RS ARE GATHERED IN THE DEVELOPING BRAIN

To contribute to neuronal development, GABA_B_-Rs must be activated by ambient GABA. Early in development, GABA is released from growth cones (Taylor and Gordon-Weeks, 1991; Gao and Van den Pol, 2000) or in paracrine non-vesicular manner (Demarque et al., 2002) providing an endogenous and local source of GABA. Using a microchemotaxis assay, Behar et al. (2001) have shown that E18 cortical plate (cp) neurons release GABA and taurine, creating a gradient that direct cell migration via the GABA_A_-Rs and GABA_B_-Rs. When GABAergic synapses are established, released GABA must diffuse to activate extra-synaptic and/or peri-synaptic GABA_B_-Rs. Consequently, GABA_B_-R mediated responses are induced when a population of interneurons are synchronously activated or facilitated when the GABA transporters are blocked (Dutar and Nicoll, 1988; Thompson and Gahwiler, 1992b; Isaacson and Nicoll, 1993; Scanziani, 2000), both procedures promoting GABA spillover. One characteristic feature of developing neuronal networks is the presence of a primitive network-driven synaptic activity both *in vivo* and *in vitro* (reviewed in Khazipov and Luhmann, 2006; Ben-Ari et al., 2007). During this patterned activity, GABAergic interneurons fired synchronously (Khazipov et al., 1997; Khazipov and Luhmann, 2006), a condition that allows diffusion of GABA into the extracellular space. This diffusion is facilitated by the presence of a large extracellular space and a relative immaturity of the reuptake mechanisms (Caillard et al., 1998; Demarque et al., 2002). Combined with synchronous release of GABA, these immature features create the adequate conditions for GABA spillover and activation of GABA_B_-Rs in the developing brain.

Applications of specific antagonists have been used to reveal the activation of GABA_B_-Rs by ambient GABA. Application of GABA_B_-R antagonists reduces the incidence of calcium spikes in the embryonic *Xenopus* spinal cord (Root et al., 2008) and increases the basal level of cAMP in embryonic rodent cortex (Bony et al., 2013). In the developing rat hippocampus, the GABA_B_-R antagonists prolong the duration of the network-driven synaptic activity, termed giant depolarizing potentials (GDPs; Ben-Ari et al., 1989; McLean et al., 1996). A similar lengthening of GDPs was observed in newborn GABA_B1_^-^^/^^-^ mice (Fiorentino et al., 2009) or after desensitization of GABA_B_-Rs following prolonged activation with baclofen (Tosetti et al., 2004, 2005). Interestingly, when the GDPs are blocked, the GABA_B_-R antagonist has no effect on GABAergic and glutamatergic synaptic activity (Colin-Le Brun et al., 2004; Fiorentino et al., 2009), supporting the idea that synchronous activation of GABAergic neurons (Khazipov et al., 1997), and subsequent GABA spillover, is required for GABA_B_-R activation. A similar GABA_B_-R dependent control of network-driven synaptic activity has been observed in developing retina (Catsicas and Mobbs, 2001), hypothalamus (Obrietan and Van den Pol, 1998, 1999), and cortex (Obrietan and Van den Pol, 1999; Kirmse and Kirischuk, 2006). In all these structures, the application of GABA_B_-R antagonists increase the frequency and/or duration of synaptic-driven intracellular calcium oscillations, showing that synaptically released GABA exerts a tonic inhibitory control of ongoing synaptic activity acting through GABA_B_Rs.

Altogether, these observations show that ambient GABA activates GABA_B_-Rs early in development, a prerequisite for a possible contribution of GABA_B_-Rs to neuronal development.

## NEUROTROPHIC ACTIONS OF GABA_B_-RS

### WHEN GABA_B_-RS MODULATE NEURONAL MIGRATION

**FIGURE 1:**
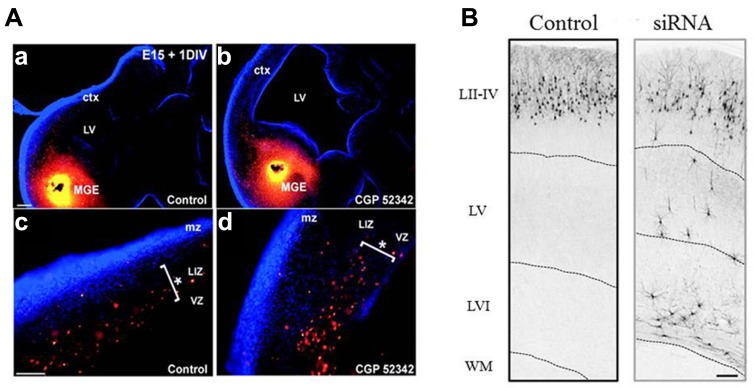
**GABA**_**B**_-Rs modulate radial and tangential migration. **(A)** E15 brain slices were cut at 300 μm and 4-chloromethyl benzoyl amino tetramethyl rhodamine (CMTMR)-coated particles were placed in the medial ganglionic eminence (MGE) of each hemisphere to label the tangentially migratory neurons. The slices were then incubated for 24 h *in vitro* in normal culture medium (a,c) or in the presence of 1 μM of the GABA_BR_ antagonist, CGP52432 (b,d). After the incubation period, the slices were fixed and counterstained with 0.002% bis-benzimide (blue) to reveal the different cortical compartments and brain structures. In control conditions more CMTMR-labeled cells located at the lower intermediate zone (LIZ) were observed (c) while in the presence of CGP52432 the majority of CMTMR-labeled cells were observed in the ventricular/subventricular zones (VZ/SVZ; d). Asterisks at (c,d) represent where the majority of the CMTMR-positive cells were localized. Abbreviations: ctx, cortex; LIZ, lower intermediate zone; mz, marginal zone; VZ, ventricular zone. Scale bars: a,b 200 μm, c,d 100 μm. Modified with permission from Lopez-Bendito et al. (2003). **(B)** Confocal images of tdTomato fluorescence in coronal sections of rat somatosensory cortices P16 after *in utero* transfection (at E17) with pRNAT-U6.3 (EGFP) siRNA empty vector together with pCAG-IRES-tdTomato (Tomato, Control), or functional GABA_B2_-siRNA (siRNA) together with Tomato construct. Dotted white lines delimitate the boundaries of the cortical layers. Note the presence of ectopic neurons that did not complete radial migration in the layers V and VI. Scale bar: 150 μm. LII–LIV, layer II–IV; WM, white matter. Modified with permission from Bony et al. (2013).

After their last division, immature postmitotic neurons migrate away from the germinal layers to reach their final position where they differentiate and establish appropriate synaptic connections. Among the many factors involved (Manent et al., 2011), GABA_B_-Rs have been reported to modulate migration or motility of embryonic spinal cord neurons (Behar et al., 1994, 1995), embryonic hypothalamic neurons (Davis et al., 2002; McClellan et al., 2008), and oligodendrocytes (Luyt et al., 2007). However, the best documented contributions of GABA_B_-Rs to neuronal migration have been obtained from the studies performed in the cortex. Two modes of migration coexist in the cortex: a radial migration of glutamatergic pyramidal cells from the ventricular/subventricular zone (VZ/SVZ) to the cp and a tangential migration of GABAergic neurons from the ganglionic eminences to the cp. The cortical VZ, cp, and tangentially migrating neurons express GABA_B_ receptors (Behar et al., 2001; Lopez-Bendito et al., 2003; Bony et al., 2013) and both radial and tangential migration are modulated by GABA_B_-Rs (**Figure 1**). The first evidence that GABA_B_-Rs modulate cortical cell migration have been obtained on dissociated embryonic cortical neurons. Starting from E15, GABA induces both chemotaxis (directed migration along a chemical gradient) and chemokinesis (random motility; Behar et al., 1996, 1998). These effects are mimicked by GABA_B_-R agonists, prevented by GABA_B_-R antagonists and suppressed by pertussis toxin, an inhibitor of G_o/i_ proteins (Behar et al., 1998). Interestingly, both migratory behaviors are eliminated when the cells are loaded with the calcium chelator BAPTA-AM [1,2-bis(2-aminophenoxy)ethane-N,N,N′,N′-tetraacetic acid tetrakis(acetoxymethyl ester)], indicating that cytosolic levels of calcium are important for GABA-induced cell motility. Accordingly, baclofen elevates intracellular calcium concentration in a subset of cells derived from E17 cortices (Behar et al., 1996). Using microdissection technique, Behar et al. (1998) have further shown that GABA_B_-Rs stimulates motility of glutamic acid decarboxylase (GAD)-positive cp neurons and directs a subset of GAD-negative VZ neurons to migrate, suggesting that these receptors may have a role in modulating radial and tangential modes of migration. Organotypic slices cultures and treatment with different GABA receptor antagonists were used to show that endogenous released GABA directs cortical cells to migrate. Thus, organotypic slice cultures treated with the GABA_B_-R antagonist CGP52342 display an accumulation in the VZ/SVZ of tangentially migrating neurons originating from the ganglionic eminences (**Figure 1A**; Lopez-Bendito et al., 2003). Similarly, treatment with the GABA_B_-R antagonist saclofen prevents most VZ BrdU-positive postmitotic neurons from entering into the cp (Behar et al., 2000). Pharmacological approaches show that GABA acts on both metabotropic and ionotropic GABA-Rs to direct neurons to their final position in the cp: GABA_C_-Rs signal cells to leave the VZ/SVZ and enter the iz; the GABA_B_-Rs direct cells to leave the iz to enter in the cp; and the GABA_A_-Rs provide a stop signal as the cells approach their target destination in the cp (Behar et al., 2000).

A recent study has addressed the role of GABA_B_-Rs on neuronal migration *in vivo* (Bony et al., 2013)*.* To this aim, short interfering RNAs (siRNAs) targeting the GABA_B2_ subunit have been electroporated at E15 to silence GABA_B_-Rs mediated responses in cortical pyramidal neuron progenitors (Bony et al., 2013). Most of the GABA_B2_-silenced cells do not reach their target normal target position in the cortical layer II/III but remained in the deep layers at postnatal stages, indicating that ambient activation of GABA_B_-Rs modulate the radial migration *in vivo* (**Figure 1B**). The authors have gathered evidence identifying the cAMP/liver kinase B1 (LKB1) signaling as downstream effector of GABA_B_-Rs (Bony et al., 2013). Indeed, the silencing of the GABA_B2_ subunit increases the immuno-staining against the phosphorylated form of LKB1 at the protein kinase A (PKA) site (pLKB1). Moreover while the overexpression of both LKB1 and constitutively active pLKB1 results in similar migration defects, the overexpression GABA_B_-Rs only rescues the LKB1-induced defects, indicating that these receptors modulate LKB1 activity through the cAMP/PKA pathway. Finally, the silencing of LKB1 expression rescues the migration defects induced by GABA_B2_ down expression *in vivo*. Altogether these data indicate that GABA_B_-Rs affect neuronal migration by modulation of cAMP/LKB1 pathway *in vivo*.

If endogenous GABA directs the migration of cortical neurons, an endogenous source of GABA must exist. Because GABA exerts chemo-attractant effect and directs migration of cortical VZ neurons into the cp, a likely candidate for the endogenous source of GABA is the cp cells. To test this hypothesis, dissociated VZ cortical cells were placed in the upper half of a chemotaxis chamber opposite to cp neurons placed in the lower half (Behar et al., 2001). In this condition, the cp cells induce the VZ cells to migrate, an effect prevented by the GABA_B_-R antagonist saclofen. This observation suggests that cp cells release a diffusible signal that stimulates migration. In agreement with this hypothesis, high performance liquid chromatography (HPLC) analysis reveals that cp neurons release GABA and taurine, and that both molecules stimulate VZ migration in a GABA_B_-R dependent manner (Behar et al., 2001). More recent studies have identified the tangentially migrating interneurons as the possible source of released GABA (Manent et al., 2005; Bony et al., 2013).

### WHEN GABA_B_-RS MODULATE CELL DIFFERENTIATION

Proper neuronal specification and acquisition of correct neurotransmitter phenotype is crucial for the functioning of the nervous system. Knockdown of GAD, the GABA synthetizing enzyme, has been used in *Xenopus* embryos to investigate the contribution of GABA in this process (Root et al., 2008). The silencing of GAD results in a decreased incidence of GABA and glycine immuno-reactive cells and increased incidence of glutamate and acetylcholine (Ach) immuno-reactive cells in the *Xenopus* spinal cord. Agarose beads loaded with a GABA_B_-R antagonist and implanted in the neural tube pheno-copied the effects of GAD knocking down. With the observation that both the GABA_B1_ and GABA_B2_ subunits are detectable in the brain and spinal cord at the corresponding developing stage (Kaeser et al., 2011), these data show that ambient GABA may control neurotransmitter specification in the *Xenopus* embryos *in vivo*. The mechanisms involved are not entirely known but required the activation of PKA and PKC which in turn stimulate generation of calcium spikes in embryonic spinal neurons (Root et al., 2008).

### WHEN GABA_B_-RS MODULATE NEURITIC OUTGROWTH AND GROWTH CONE GUIDANCE

To ensure accurate targeting, neurons use different regulated mechanisms that provide growth and directional cues to advancing growth cones. Among the many factors, a large amount of evidence indicates that GABA can modulate neurite outgrowth *in vitro* in different brain regions and species (for review, see Sernagor et al., 2010). Although most studies have focused on the contribution of GABA_A_-Rs, there are some indications that GABA_B_-R activation also modulates neuritic growth and growth cone motility both *in vitro* and *in vivo*.

The first evidence that GABA_B_-Rs may affect neurite outgrowth was provided by Michler (1990). They have shown that the administration of the GABA_B_-R agonist baclofen to cultures obtained from chick tectum or rat cerebellum inhibits neurite outgrowth, while administration of the GABA_B_-R antagonist saclofen leads to opposite effect (Michler, 1990). Similar to the earlier study by Michler (1990), baclofen has been reported to inhibit the neurite outgrowth and growth cone motility of mice spinal cord (Bird and Owen, 1998) and olfactory bulb (Priest and Puche, 2004) neurons kept in cultures. GABA_B_-Rs also modulate the neurite outgrowth of cultured cortical neurons (Bony et al., 2013). Indeed, cortical neurons treated with baclofen exhibit longer and more branched dendrites associated with shorter and less branched axons. Conversely, treatment with the GABA_B_-R antagonist CGP55845 results in shorter dendrites and longer axons. Interestingly, siRNA transfection to silent the expression of the GABA_B2_ subunit also shortens dendritic length and promotes axonal length (Bony et al., 2013). With this approach, the post-synaptic GABA_B_-R mediated responses were silenced in a small population of neurons that grow in a normally developing network, hence ruling out impact on the overall network activity, in contrast to the pharmacological approach in which the pre- and post-synaptic responses were abolished in every cell. Thus, the neurotrophic action of GABA_B_-Rs is not an indirect consequence of altered synaptic activity, but rather results from the activation of post-synaptic downstream signaling pathways. GABA_B_-Rs not only modulate neuritic growth of some neuronal populations, they also modulate axon/dendrite polarity of cortical neuron *in vitro*. Baclofen inhibits axon initiation whereas CGP55845 or GABA_B2_ silencing promote axon initiation *in vitro* (Bony et al., 2013). The effects of baclofen and CGP55845 are abolished by, respectively, an activator or an inhibitor of the adenylate cyclase, indicating that GABA_B_-Rs modulate neuronal polarity through an inhibition of cAMP signaling. Gascon et al. (2006) have examined the effects of GABA signaling on dendritic development *in vitro* using purified neurons from the SVZ of newborn rats, intended to the olfactory bulb neurons. They found that although cells treated with the GABA_A_-R antagonist bicuculline exhibit reduced dendritic complexity, treatment with a GABA_B_-R antagonist has no significant effects. Similarly, blockade of GABA_B_-R does not affect the neuritic outgrowth of cultured cp neurons (Maric et al., 2001). These findings may suggest that either the modulation of neurite outgrowth exerted by the GABA_B_-Rs is cell type specific or that in these cultures the amount of GABA released do not reach the critical concentration allowing the activation of extra/peri-synaptic GABA_B_-Rs.

Despite a large body of data indicating a role for GABA_B_-Rs in neurite outgrowth *in vitro*, *in vivo* evidence have been provided only recently. In the *Xenopus laevis*, the axons of retinal ganglion cells (RGCs) extend through the brain toward their major target, the optic tectum. The axon growth cones of RGCs express GABA_B_-Rs, and application of baclofen stimulate RGC neurite outgrowth in cultures (Ferguson and McFarlane, 2002). To determine the contribution of the GABA_B_-Rs *in vivo*, Ferguson and McFarlane (2002) have applied the GABA_B_-R antagonist CGP54626 and found that it causes a shortening of the optic projections, indicating that ambient activation of GABA_B_-Rs controls the growth of RGC axons *in vivo*. To investigate whether GABA_B_-Rs may also modulate the neurite outgrowth of cortical pyramidal neurons *in vivo*, siRNA were electroporated *in utero* to downregulate the expression of the GABA_B2_ subunit to silence post-synaptic GABA_B_-R mediated responses (Bony et al., 2013). Morphological analysis revealed that the specific downregulation of endogenous GABA_B2_ subunit *in vivo* leads to reduced apical dendritic length and branch number, and increased axonal growth of cortical neurons. In contrast, the dendritic length and complexity of hippocampal CA3 pyramidal neurons is not altered in GABA_B1_^-^^/^^-^ knockout mice (Fiorentino et al., 2009). This observation may suggest that GABA_B_ receptors regulate neurite outgrowth in some, but not all, brain regions or, that compensatory mechanism take place in the knockout mice (see below).

If GABA_B_-Rs can modulate neurite outgrowth, an obvious question was to determine whether they could also serve as guidance signal for axon pathfinding. To answer that question, Xiang et al. (2002) applied a gradient of baclofen to *Xenopus* spinal cord neurons in cultures. They found that the growth cones turn away from the baclofen gradient. Interestingly, repulsion is converted to attraction in the presence of PKC inhibitors. Pharmacological investigations show that baclofen exerts a bi-directional control of growth cone guidance through the activation of different signaling pathways: the activation of the PLC–PKC pathway leading to growth cone repulsion and the PLC-IP3 pathway that attracts the growth cone toward the baclofen source (Xiang et al., 2002). Whether and which of these signaling pathways contribute to axon guidance *in vivo* remains to be elucidated.

### WHEN GABA_B_-RS MODULATE SYNAPTOGENESIS

**FIGURE 2:**
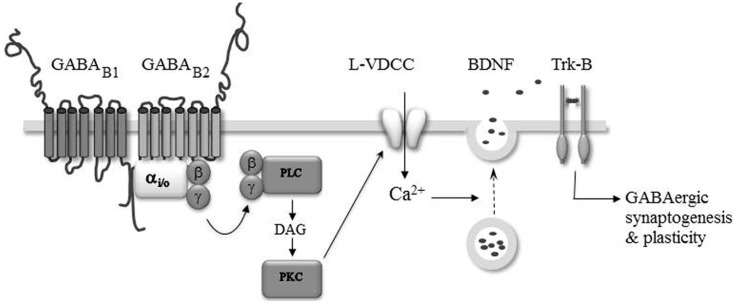
**GABA**_**B**_-Rs modulate GABAergic synaptogenesis and plasticity. The activation of GABA_B_-Rs induces the formation of diacylglycerol (DAG), activation of protein kinase C (PKC), and the opening of L-type voltage-dependent Ca^2^^+^ channels (L-VDCC). The intracellular Ca^2^^+^ rise triggers the secretion of BDNF which acting on TrkB-Rs modulate the formation and efficacy of developing GABAergic synapses.

The above gathered studies indicate that activation of GABA_B_-Rs could serve as a growth and guidance signal, thereby participating in neuronal network construction. Recent evidence indicates that GABA_B_-Rs may also regulate synaptogenesis. Chattopadhyaya et al. (2007) demonstrated in organotypic cortical slices that endogenous GABA regulates the axonal branching of basket-cell interneurons through the activation of GABA_A_ and GABA_B_ receptors. The authors found that knocking down GAD67, the major GABA synthetizing enzyme, in single basket cells results in a cell autonomous decrease in the number of peri-somatic GABAergic synapses formed by these interneurons on pyramidal cells. The beauty of this approach is that a single cell manipulation minimally impacts the overall level of synaptic activity in contrast to pharmacological manipulations. The deficit induced by the down expression of GAD67 is partially rescued by adding inhibitors of GABA uptake or GABA_A_ as well as GABA_B_ receptor agonists. These findings therefore indicate that ambient GABA, acting on both GABA_A_ and GABA_B_ receptors, contributes to the development of peri-somatic cortical inhibition. In line with GAD silencing, knockout of the GABA_B1_ subunit also results in altered development of GABAergic synaptic transmission in the mice hippocampus (Fiorentino et al., 2009). Whole cell recordings performed on acute hippocampal slices obtained from newborn GABA_B1_^-^^/^^-^ mice reveals a lower level of miniature GABAergic synaptic activity in the mutant CA3 pyramidal neurons compared to the WT. This deficit is reproduced *in vitro* in newborn WT intact hippocampi incubated overnight with the GABA_B_-R antagonist CGP55845 or with tetrodotoxin (TTX) to block action potential-dependent synaptic activity. Importantly, the deficit induced by TTX treatment is rescued by baclofen, indicating that synaptic activation of GABA_B_-Rs by ambient GABA contributes to the functional development of hippocampal GABAergic synapses. The mechanism by which GABA_B_-Rs promote the development of GABAergic synapses likely involved regulated secretion of the brain-derived neurotrophic factor (BDNF) and subsequent activation of the tropomyosin receptor kinase (TrkB) pathway (**Figure 2**). Indeed, the deficit in GABAergic activity observed in WT intact hippocampi treated overnight with CGP55845 is rescued by BDNF treatment and occluded by the absence of BDNF (i.e., in intact hippocampi obtained from BDNF^-^^/^^-^ mice or treated with the BDNF/NT4 scavenger TrkB–immunoglobulin G, IgG; Fiorentino et al., 2009). Furthermore, time lapse fluorescence imaging of BDNF–GFP expressing neurons and immuno-histochemical studies show that the activation of GABA_B_-Rs triggers a calcium-dependent secretion of BDNF *in vitro* through the activation of the phospholipase C (PLC)–PKC pathway and opening of L-type voltage-dependent calcium channels (Fiorentino et al., 2009; Kuczewski et al., 2011). A previous study has shown that the phosphorylation of the α-CamKII, a critical step in BDNF secretion (Kolarow et al., 2007), is enhanced by GABA_B_-R activation in the developing rat hippocampus (Xu et al., 2008). Therefore, post-synaptic elevation of calcium and phosphorylation of α-CamKII may underlie the GABA_B_-R induced secretion of BDNF and functional maturation of GABAergic synaptic transmission in the developing hippocampus.

Brain-derived neurotrophic factor and tropomyosin receptor kinase signaling is necessary for the full development of inhibitory circuitry (Gottmann et al., 2009). Moreover, endogenous activation of GABA_B_-Rs (McLean et al., 1996; Obrietan and Van den Pol, 1998, 1999; Catsicas and Mobbs, 2001; Kirmse and Kirischuk, 2006) and GABA_B_-R dependent modulation of intracellular calcium levels have been reported in several developing brain structures (Behar et al., 1996; Hirono et al., 2001; New et al., 2006; Root et al., 2008; Kuczewski et al., 2011). Therefore, the GABA_B_-R induced secretion of BDNF may be important for the maturation of synaptic connections throughout the nervous system. Accordingly, a GABA_B_-R and BDNF–TrkB dependent plasticity of developing inhibitory transmission has been observed in the auditory system (Kotak and Sanes, 2000, 2002; Chang et al., 2003) and visual cortex (Komatsu and Iwakiri, 1993; Inagaki et al., 2008). In these structures, the plasticity is induced during a restricted period of development suggesting that it may have a developmental function in the refinement of initially coarse patterns of synaptic connections (Katz and Shatz, 1996). How GABA_B_-R and BDNF are linked is presently not fully understood. However, based on the observation that bath application of BDNF rescues the inhibitory plasticity in neurons loaded with the calcium chelator BAPTA, it was proposed that the induction of the inhibitory plasticity relies on the endogenous activation of GABA_B_-Rs leading to a calcium-dependent secretion of BDNF from the target neurons, which through the TrkB pathway triggered synaptic plasticity (Inagaki et al., 2008; **Figure 2**).

## THE INCONSISTENCY OF GABA_B_-R KNOCKOUT MICE

Although *in vitro* pharmacological studies indicate that GABA_B_-Rs can function as a development signal, studies of knockout mice lacking functional GABA_B_-Rs could lead to opposite conclusions. Indeed, the complete silencing of GABA_B_-Rs in mice causes behavioral alterations such as epilepsy, impaired memory, hyperalgesia, hyperthermia, and hyperactivity, highlighting the importance of GABA_B_-Rs in the appropriate functioning of the nervous system (Prosser et al., 2001; Queva et al., 2003; Haller et al., 2004; Wu et al., 2007). Yet, knockout of either GABA_B1_ or GABA_B2_ subunit does not reveal significant alteration in cortical layer organization, although a role of GABA_B_-Rs in cortical migration has been identified *in vitro*. Similarly, morphological analysis of hippocampal pyramidal neurons reveals no significant alteration of GABA_B1_^-^^/^^-^ mice (Fiorentino et al., 2009), although endogenous GABA_B_-Rs have been reported to modulate neurite growth *in vitro* (Michler, 1990; Priest and Puche, 2004; Bony et al., 2013). The same discrepancy is observed in mice knockout for the GABA-synthetizing enzyme GAD. Indeed, although GABA is scarcely detected in double knockout GAD 65 and 67, no disorders of histogenesis are observed (Ji et al., 1999). It should be mentioned, however, that a developmental phenotype has been observed in GABA_B1_^-^^/^^-^ mice, in which whole cell recordings of hippocampal slices reveal a delayed functional maturation of GABAergic synapses in the mutant mice (Fiorentino et al., 2009). Therefore, a more detailed determination of the morphological–functional properties of the neuronal networks in GABA_B_^-^^/^^-^ mice is essential to ensure that the mutation has developmental consequences. The discrepancy between *in vitro* pharmacological studies and genetically modified mice *in vivo* may be explained by compensatory mechanisms frequently experienced by knockout mice. For instance, in the knockout GAD mice, taurine can bind GABA_B_-Rs and could potentially compensate for the absence of GABA (Behar et al., 2001). Moreover, some of the downstream signaling pathways activated by the GABA_B_-Rs are also the targets of other receptors, and activation of these receptors may substitute for the absence of functional GABA_B_-Rs. A recent study shows that silencing GABA_B_-Rs in a small population of excitatory neurons leads to ectopic positions and morphological alterations of these neurons (Bony et al., 2013), hence highlighting the contribution of GABA_B_-Rs to neuronal development *in vivo*.

## CONCLUSION

A large body of studies has documented the prominent role of GABA_A_-Rs to neuronal development. For several years, the possible contribution of GABA_B_-Rs has lagged behind. The studies gathered in this review indicate that GABA_B_-Rs do play a role in crucial steps of neuronal network formation, including migration, neurite growth, synapse formation and plasticity, both *in vitro* and *in vivo* (**Table 1**). The neurotrophic action of GABA_B_-Rs raises several immediate questions. Do they play an instructive or a permissive role? What are the downstream signaling pathways involved? Does the use pharmacological drugs during pregnancy lead to GABA_B_-dependent alterations in brain construction?

**Table 1:** Neurotrophic action of GABA_**B**_-Rs

Structure	Preparation	Approach to silence GABA_B_-Rs	Consequences of GABA_B_-Rs silencing	Reference
Rat neocortex	Dissociated neuronal cultures	GABA_B_-R antagonist	Decrease radial migration	Behar et al. (1996, 1998)
Rat neocortex	Organotypic slice cultures	GABA_B_-R antagonist	Decrease tangential migration	Lopez-Bendito et al. (2003)
Mice neocortex	*In* *vivo*	In utero GABA_B2_ silencing	Decrease radial migration and neurite outgrowth	Bony et al. (2013)
*Xenopus* spinal cord**	*In* *vivo*	GABA_B_-R antagonist	Inhibit phenotypic differentiation of GABA and glycine cells	Root et al. (2008)
Chick tectum	Dissociated neuronal	GABA_B_-R antagonist	Inhibit neurite outgrowth	Michler (1990)
Rat cerebellum	cultures			Priest and Puche (2004)
Mice olfactory bulb				Bird and Owen (1998)
Mice spinal cord				Bony et al. (2013)
Rat neocortex				
*Xenopus* spinal cord**	Dissociated neuronal cultures	GABA_B_-R antagonist	Modulate growth cone guidance	Xiang et al. (2002)
*Xenopus* retinal ganglion cell**	*In* *vivo*	GABA_B_-R antagonist	Inhibit axon outgrowth	Ferguson and McFarlane (2002)
Mice hippocampus	Acute slices	GABA_B1_^-^^/^^-^ knockout mice	Decrease GABAergic synaptic maturation	Fiorentino et al. (2009)
Rat visual cortex	Organotypic slice cultures	Single cell GAD knockout	Decrease GABAergic synaptogenesis	Chattopadhyaya et al. (2007)

The use of antagonists has been extremely fruitful to unmask the endogenous activation of GABA_B_-Rs and reveal their neurotrophic actions. However, because GABA_B_-Rs control the level of synaptic activity, it is often difficult with this approach to distinguish between indirect actions on synaptic activity from direct consequences of GABA_B_-R activation. However, the functional silencing of GABA_B_-Rs in a small population of neurons that minimally impact the level of synaptic activity offers an interesting alternative, and confirms that endogenous GABA_B_-Rs exert a neurotrophic action on neuronal development *in vivo*. Some signaling pathways have been identified, involving changes in calcium and cAMP levels, but much remained to be done. *In vitro* studies have shown that the stimulation of GABA_B_-Rs leads to a translocation of the transcriptor factor 4 (ATF4; White et al., 2000; Vernon et al., 2001) and to the phosphorylation cAMP-response element binding (CREB). The interactions with ATF4 and/or CREB may be important to regulate gene expression and may underlie some of the neurotrophic actions of the GABA_B_-Rs. Accordingly, the direct activation of GABA_B_-Rs, in the presence of TTX to block spontaneous synaptic activity, leads to an up-regulation of BDNF expression, a key modulator of neuronal network wiring (Gottmann et al., 2009).

A recent study has revealed that patients with autoimmune encephalitis associated with antibodies to GABA_B1_ subunits show seizures, confusion and memory deficit (Lancaster et al., 2010). Moreover, some GABA_B_-R polymorphisms confer a highly increased susceptibility to temporal-lobe epilepsy in human beings (Gambardella et al., 2003; Wang et al., 2008). Thus, defective GABA_B_-Rs functions in the brain could underlie neurological and psychiatric diseases. It is therefore tempting to speculate that children exposed to drugs that affect GABA levels or act on GABA_B_-Rs during their fetal or postnatal life may develop GABA_B_-R related developmental disorders. In future studies, it will be essential to consider the emerging role of GABA_B_-Rs in network formation to fully understand the neurotrophic role of GABA and pathological consequences of altered GABAergic signaling.

## Conflict of Interest Statement

The authors declare that the research was conducted in the absence of any commercial or financial relationships that could be construed as a potential conflict of interest.
